# Teaching Anti-Racism in Gerontology: An Interactive Program of Recognition, Self-Work, Pedagogy, and Action

**DOI:** 10.1093/geroni/igab046.469

**Published:** 2021-12-17

**Authors:** Althea Pestine-Stevens, Tina K Newsham

## Abstract

Older adults with intersecting identities as persons of color experience disparities in health and well-being due to racism in individual and structural spheres, which have been amplified by health, economic, and social consequences of COVID-19. We can begin the work to reduce these inequities by training scholars and practitioners to disrupt the systems within which we work that relegate advantages and disadvantages throughout the life course and in later life by racial groups. This interactive symposium presents resources on anti-racist gerontological education and provides an opportunity to engage critically with peers in all stages of their careers and anti-racism journeys who are interested in integrating anti-racism into their teaching. The first presenter introduces conversations to begin anti-racist pedagogy and assumptions to dismantle. The second presenter describes cultural humility as an essential step towards self-awareness and critical self-reflection for educators and practitioners. The third presenter presents how anti-racist pedagogy, a teaching approach that combines racial content, pedagogy, and organizing, may be applied to gerontology education. Fourth, an example will be presented from an online course module developed to guide Master of Social Work students toward recognizing racial disparities in aging services systems and identifying concrete suggestions for improvement. Finally, strategies for curriculum design will be presented with examples from Public Health education. This symposium is designed to include ample time for group discussion on this critical and under-addressed area of teaching in gerontology across disciplines, such that participants can better connect with others to build awareness, competency, and resources.

